# Prognostic Significance of CD47 and DHX9 in Metastatic Rectal Cancer

**DOI:** 10.3390/medsci14060637

**Published:** 2026-10-03

**Authors:** Sendag Yaslikaya, Suheda Atas Ipek, Tugba Toyran, Ertugrul Bayram, Ismail Oguz Kara

**Affiliations:** 1 Department of Medical Oncology, Giresun Training and Research Hospital, 28100 Giresun, Turkey; 2 Department of Medical Oncology, Faculty of Medicine, Cukurova University, 01330 Saricam, Turkey; 3 Department of Pathology, Faculty of Medicine, Cukurova University, 01330 Saricam, Turkey

**Keywords:** rectal cancer, CD47, DHX9, prognosis, overall survival, immunohistochemistry

## Abstract

**Background/Objectives:** The prognosis of metastatic rectal cancer remains poor, and few reliable prognostic biomarkers are available. DHX9 and CD47 have been implicated in colorectal cancer biology, but their prognostic value in metastatic disease remains unclear. We evaluated the associations of DHX9 and CD47 expression with clinical characteristics, molecular markers and overall survival. **Methods:** This retrospective, single-center cohort study included 65 patients with metastatic rectal cancer treated between 2010 and 2023. Expression of DHX9 and CD47 in primary tumor tissue was assessed by immunohistochemistry and classified as low or high. Clinical characteristics, metastatic presentation (de novo or recurrent), metastatic sites and molecular markers (KRAS, NRAS, BRAF and HER2) were recorded. Associations with marker expression were examined. Overall survival was analyzed using the Kaplan–Meier method, log-rank test and multivariable Cox proportional hazards regression adjusted for clinical and molecular factors. **Results:** High DHX9 expression was observed in 87.7% of tumors and high CD47 expression in 35.4%. During a median follow-up of 24 months, 53.1% of patients died. High CD47 expression was associated with shorter overall survival (log-rank *p* = 0.016) and this association persisted after adjustment for age, sex, metastatic presentation and DHX9 expression (HR = 2.71; 95% CI 1.34–5.48; *p* = 0.006). The association remained significant after further adjustment for RAS/RAF mutation status and HER2 positivity (HR = 2.78; 95% CI 1.02–7.59; *p* = 0.047). DHX9 expression was not associated with overall survival (log-rank *p* = 0.243). Expression of neither marker differed between de novo and recurrent metastatic disease or according to RAS, BRAF or HER2 status. **Conclusions:** In this exploratory, single-centre analysis, high CD47 expression was associated with shorter overall survival, whereas DHX9 expression was not. These hypothesis-generating findings require confirmation in larger, prospective, multicentre studies before any clinical application.

## 1. Introduction

Rectal cancer remains a leading contributor to cancer morbidity and mortality worldwide, and many patients progress to metastatic disease over the course of illness [1,2]. Prognosis in metastatic disease is poor, with the liver being the predominant metastatic site, followed by the lungs and peritoneum [2]. Although outcomes have improved with modern systemic therapy, survival varies widely and is strongly influenced by tumor biology, including RAS and BRAF mutation status, as well as by the pattern and extent of metastatic spread [3]. Established clinical and molecular factors do not fully explain this variation, and additional biomarkers are needed to refine prognostic assessment in metastatic disease.

DHX9 (RNA helicase A) is an NTP-dependent helicase that participates in DNA replication, transcription and RNA processing [4]. Its overexpression has been reported across a range of tumour types and associated with enhanced proliferation, invasion, metastatic capacity and worse outcomes [5]. In colorectal cancer, DHX9 promotes tumor progression partly through the activation of NF-κB signaling, with its upregulation also associated with poor clinical outcomes [6]. These findings support a potential prognostic role, but the prognostic value of DHX9 in metastatic disease remains unexplored.

CD47 is a transmembrane glycoprotein that acts as a “don’t eat me” signal: by engaging SIRPα on macrophages, it suppresses phagocytosis and allows tumour cells to escape innate immune clearance [7]. Frequently overexpressed in solid tumours, CD47 has been linked in colorectal cancer to unfavourable clinicopathological features and reduced survival [8]. The CD47–SIRPα axis has also emerged as a therapeutic target, with agents that block this interaction currently under investigation in solid tumors [9]. However, the prognostic relevance of CD47 in metastatic disease remains poorly defined.

Given the limited evidence in metastatic disease, we evaluated the immunohistochemical expression of DHX9 and CD47 in primary tumor tissue from patients with metastatic rectal cancer. We examined the associations of these markers with clinical characteristics, molecular markers and overall survival.

## 2. Materials and Methods

### 2.1. Study Design

This retrospective, single-center cohort study evaluated the associations of DHX9 and CD47 immunohistochemical expression with clinical characteristics, molecular markers and overall survival in patients with metastatic rectal cancer. Ethical approval for the protocol was granted by the Çukurova University Faculty of Medicine Ethics Committee on 23 July 2023 (no. 16258), and the study followed the ethical standards set out in the Declaration of Helsinki. The present analysis was based on a prespecified metastatic subgroup drawn from a larger cohort of rectal adenocarcinoma patients of all stages assembled within the same research project.

### 2.2. Patient Population

We retrospectively reviewed patients with histopathologically confirmed metastatic rectal adenocarcinoma managed at the Department of Medical Oncology, Çukurova University Faculty of Medicine, between 2010 and 2023. Of a larger cohort of 210 patients with rectal adenocarcinoma of all stages assembled within the same research project, 74 had metastatic disease and were screened. After excluding patients with a concurrent second malignancy, non-adenocarcinoma histology, or insufficient tumour tissue for immunohistochemistry, 65 patients formed the final descriptive cohort. De novo versus recurrent disease was classified by the timing of metastasis relative to the initial diagnosis: de novo denoted metastasis present at diagnosis, and recurrent denoted metastasis developing after initially non-metastatic disease. This was based on the date of metastatic diagnosis in the records and applied uniformly to both groups. Complete follow-up data were available for 64 of the 65 patients; one patient with de novo disease had no follow-up information and was retained in the descriptive cohort (*n* = 65) but excluded from all survival analyses (*n* = 64). Data on age, sex, body mass index (BMI), metastatic presentation, metastatic sites, follow-up duration and vital status were obtained from electronic medical records. KRAS, NRAS and BRAF mutation status and HER2 expression status were extracted from pathology and molecular diagnostic reports.

### 2.3. Immunohistochemical Staining

For DHX9 and CD47, 4-µm sections were cut from formalin-fixed, paraffin-embedded primary tumour blocks and stained in the Department of Medical Pathology, Çukurova University Faculty of Medicine. Immunohistochemistry was performed on primary tumour tissue obtained at initial diagnostic biopsy or surgical resection, before any systemic or neoadjuvant therapy, so marker expression reflects treatment-naïve tissue. Staining used anti-DHX9 (rabbit monoclonal, clone EPR13521; Abcam, Cambridge, UK; ab183731; 1:100) and anti-CD47 (rabbit monoclonal, clone EPR21794; Abcam, Cambridge, UK; ab218810; 1:2000) on a Ventana BenchMark automated stainer (Roche Diagnostics, Tucson, AZ, USA), with heat-induced epitope retrieval in EDTA buffer (pH 9) for 20 min and visualisation by a polymer-based HRP system with DAB (ultraView; Ventana Medical Systems, Tucson, AZ, USA). Tonsil tissue served as positive control, and the primary antibody was omitted for negative controls. Expression of DHX9 and CD47 was independently evaluated by two experienced pathologists who were blinded to clinical characteristics and survival outcomes. For each case, the percentage of positively stained tumour cells was assessed over five representative ×200 fields (≥1000 tumour cells), with DHX9 scored as nuclear and CD47 as membranous staining. Cases were classified as high expression when ≥50% of tumour cells showed nuclear DHX9 staining and ≥25% showed membranous CD47 staining. As no standardised cut-offs exist for either marker [10], these thresholds were pre-specified by the study pathologist before survival analysis, at conventional boundaries of semi-quantitative percentage categories [11,12]. Discrepancies between the two assessments were resolved by consensus review. As each case was recorded as a single consensus score rather than two independent scores, a formal interobserver agreement coefficient was not applicable.

Patients were followed at regular intervals from the diagnosis of metastatic disease. Overall survival, the primary endpoint, was measured from the date of metastatic diagnosis to death from any cause; patients still alive at last follow-up were censored at their date of last contact. Overall survival was calculated in months.

### 2.4. Statistical Analysis

All analyses were carried out in SPSS v26.0 (IBM Corp., Armonk, NY, USA). Distributional normality of continuous measures was checked with the Kolmogorov–Smirnov test. Depending on this, continuous data are presented either as mean ± SD or, where normality did not hold, as median with minimum and maximum; counts and proportions are given for categorical variables.

Two-group comparisons of continuous variables used the *t* test or the Mann–Whitney U test according to distribution, while categorical variables were assessed with the chi-square or Fisher’s exact test. Survival was described with Kaplan–Meier estimates and compared across groups by the log-rank statistic. Multivariable Cox proportional hazards regression identified independent prognostic factors for overall survival. Model 1 included age, sex, metastatic presentation, DHX9 expression and CD47 expression. Model 2 additionally included RAS/RAF mutation status and HER2 positivity. RAS/RAF status was a composite variable. Tumours with a KRAS, NRAS or BRAF mutation were classified as mutant, and those wild-type for all tested genes as wild-type. Patients with no result for any of the three genes were treated as missing. KRAS, NRAS and BRAF were tested simultaneously using real-time polymerase chain reaction (PCR) in 49 patients; one patient with a failed KRAS result carried a BRAF mutation and was classified as mutant. HER2 was assessed by immunohistochemistry, and 3+ staining, or 2+ staining with amplification on in situ hybridisation, was considered positive. Model 2 was a complete-case analysis of the 49 patients with complete data for all model covariates. To assess dependence on dichotomisation, DHX9 and CD47 were also entered simultaneously as continuous staining percentages (per 10% increment) into a model with the same adjustment variables as Model 1 (age, sex, metastatic presentation; *n* = 64, 34 events). The assumed linear relationship between each continuous marker and the log hazard was consistent with visual inspection of Martingale residual plots. To separate the effect of adjustment strategy from the effect of sample composition, Model 1 was additionally re-run in the Model 2 complete-case subset (*n* = 49, 23 events). The proportional hazards assumption was assessed using Schoenfeld residuals and was satisfied for all covariates; the number of covariates per model was kept limited given the number of events. Cox results are expressed as HRs with accompanying 95% CIs. Two-sided *p* values below 0.05 denoted significance. The study was reported in accordance with the REMARK guidelines [13].

## 3. Results

**Figure 1:**
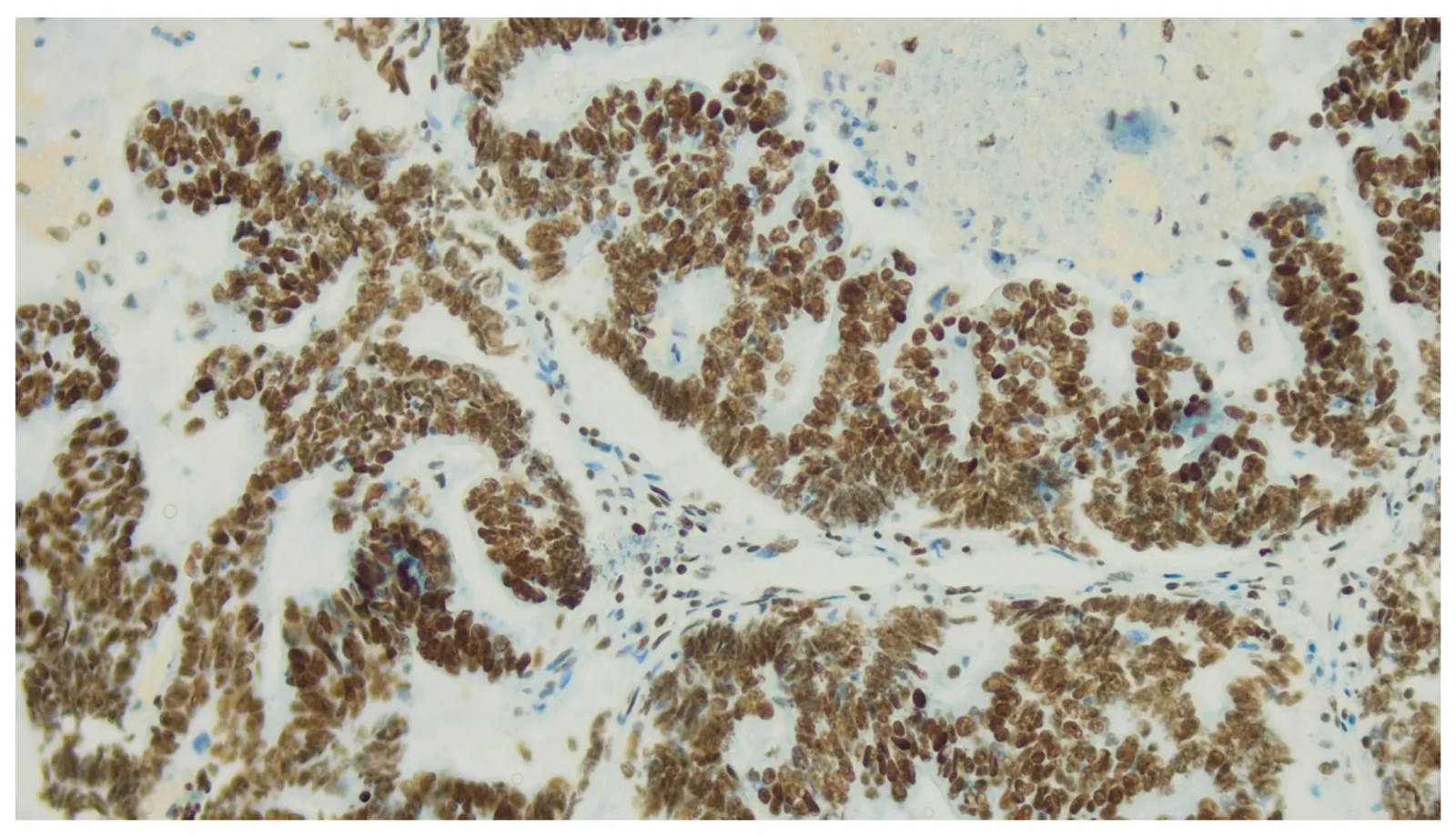
Representative immunohistochemical staining of DHX9 in rectal cancer showing nuclear positivity (×200). Brown staining indicates positive immunoreactivity.

**Figure 2:**
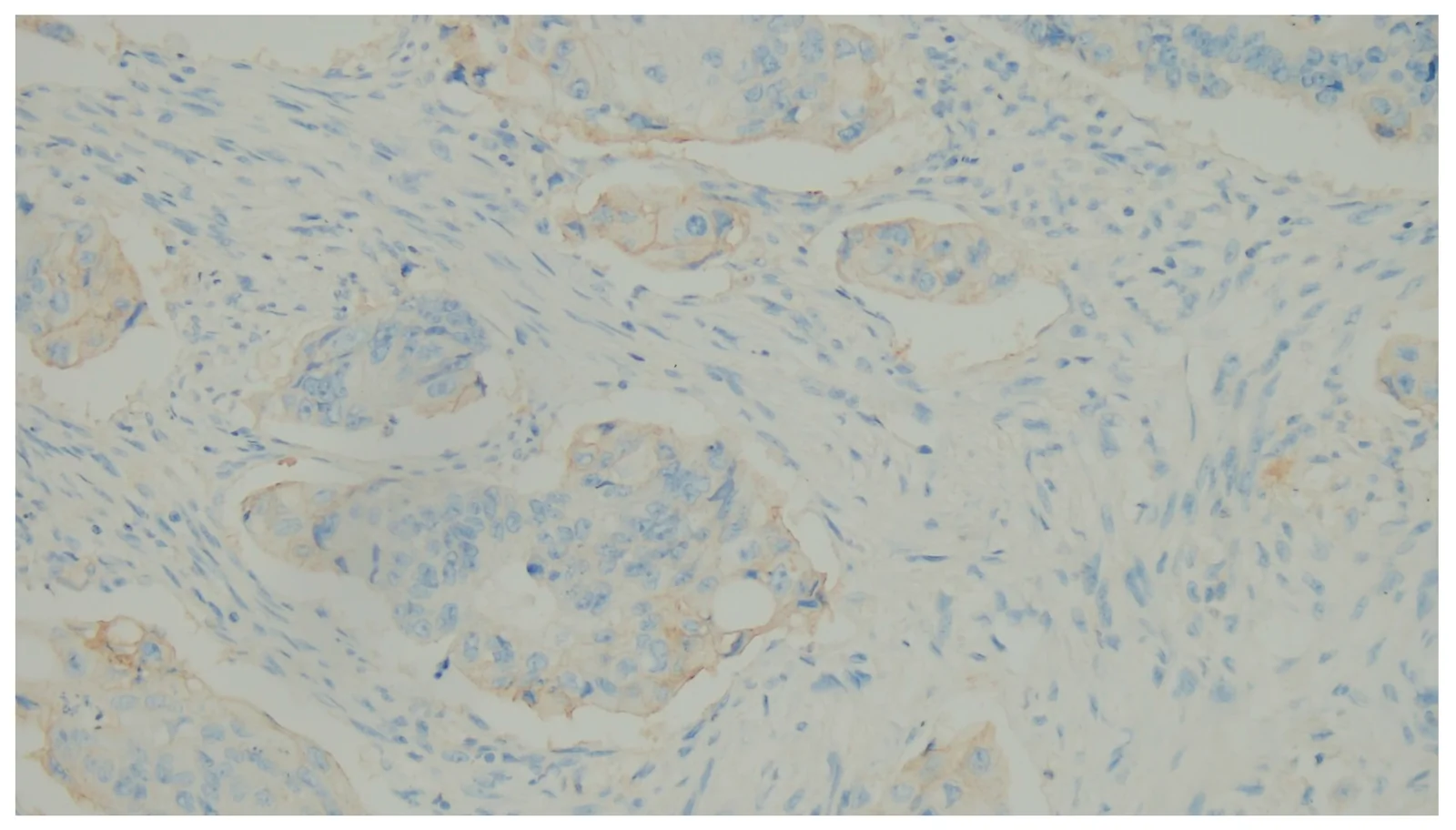
Representative immunohistochemical staining of CD47 in rectal cancer showing cytoplasmic/membranous positivity (×200). Brown staining indicates positive immunoreactivity.

Sixty-five patients with metastatic rectal cancer were analysed. Median age was 63 years (28–80), and 63.1% were men. Metastatic disease was present at diagnosis (de novo) in 40.0% of patients, while the remaining 60.0% had recurrent disease. The most common metastatic pattern was isolated liver metastasis (44.6%), followed by diffuse or multiple-organ metastatic disease (33.8%). High DHX9 expression was observed in 87.7% of tumors (Figure 1) and high CD47 expression in 35.4% (Figure 2). During a median follow-up of 24 months (range, 3–114), 53.1% of patients died; 47.7% experienced disease progression or recurrence (Table 1). Median overall survival was 28 months (95% CI 19–37), and 34 of the 64 patients with follow-up data died.

**Figure 3:**
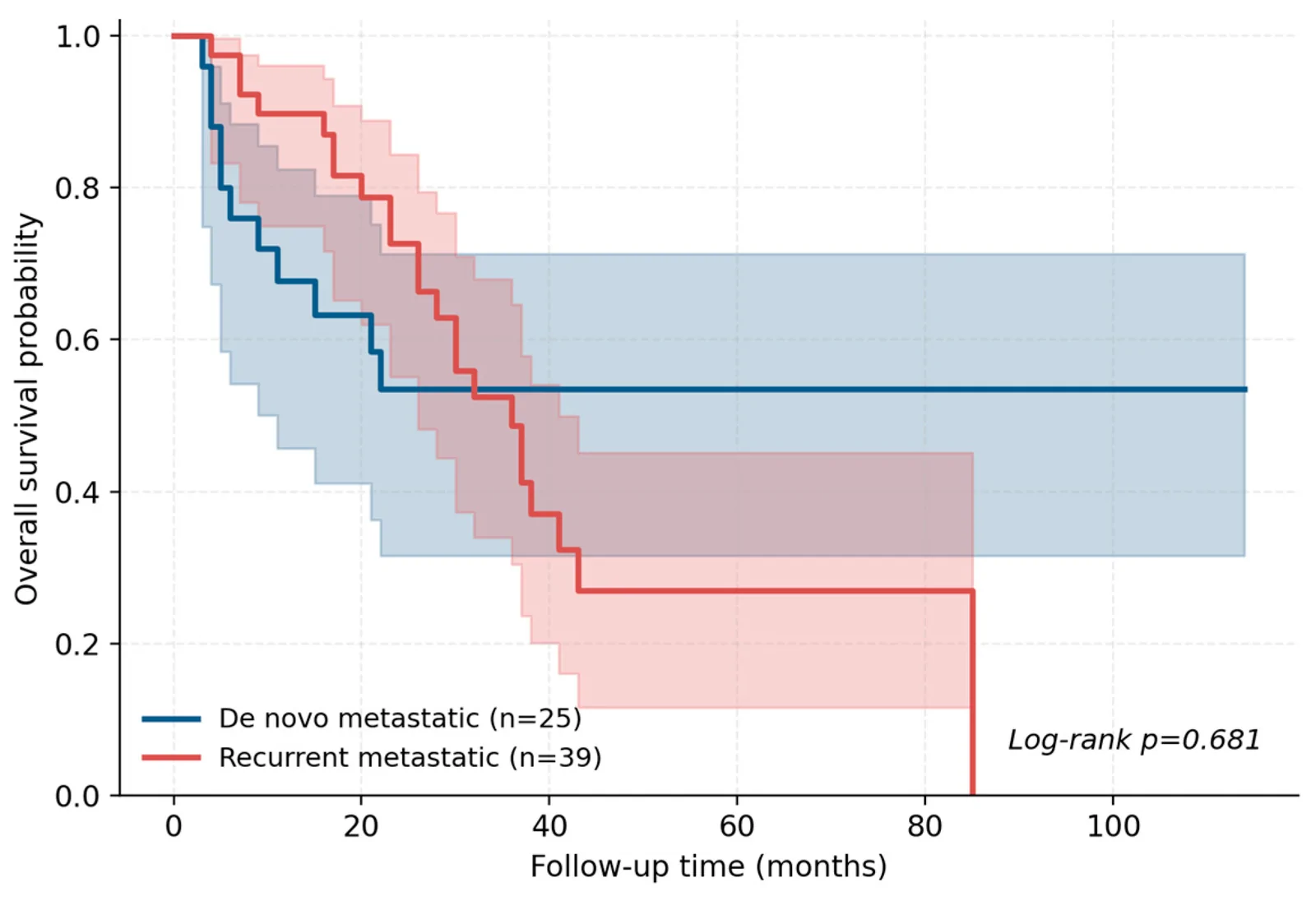
Overall survival comparison between de novo and recurrent metastatic disease (log-rank *p* = 0.681). Although the de novo group comprises 26 patients in total (Table 1 and Table 2), 1 patient with missing follow-up duration was excluded from the Kaplan–Meier analysis, so the de novo group is shown as *n* = 25 in the figure; there is no missing data in the recurrent group (*n* = 39). Shaded areas indicate 95% confidence intervals.

No significant differences were observed between patients with de novo and recurrent metastatic disease in the rates of high DHX9 expression (88.5% vs. 87.2%; *p* = 1.000) or high CD47 expression (34.6% vs. 35.9%; *p* = 1.000). Mean DHX9 staining percentages (76.5 ± 28.1 vs. 72.8 ± 29.4; *p* = 0.610) and CD47 staining percentages (24.4 ± 30.7 vs. 24.1 ± 30.1; *p* = 0.970) were also comparable between the groups. Overall survival did not differ significantly between patients with de novo and recurrent metastatic disease (log-rank *p* = 0.681) (Table 2, Figure 3).

Neither DHX9 nor CD47 expression was significantly associated with BRAF mutation, KRAS mutation or HER2 positivity (all *p* > 0.05). NRAS mutations were not detected in any of the evaluated cases (Table 3).

**Figure 4:**
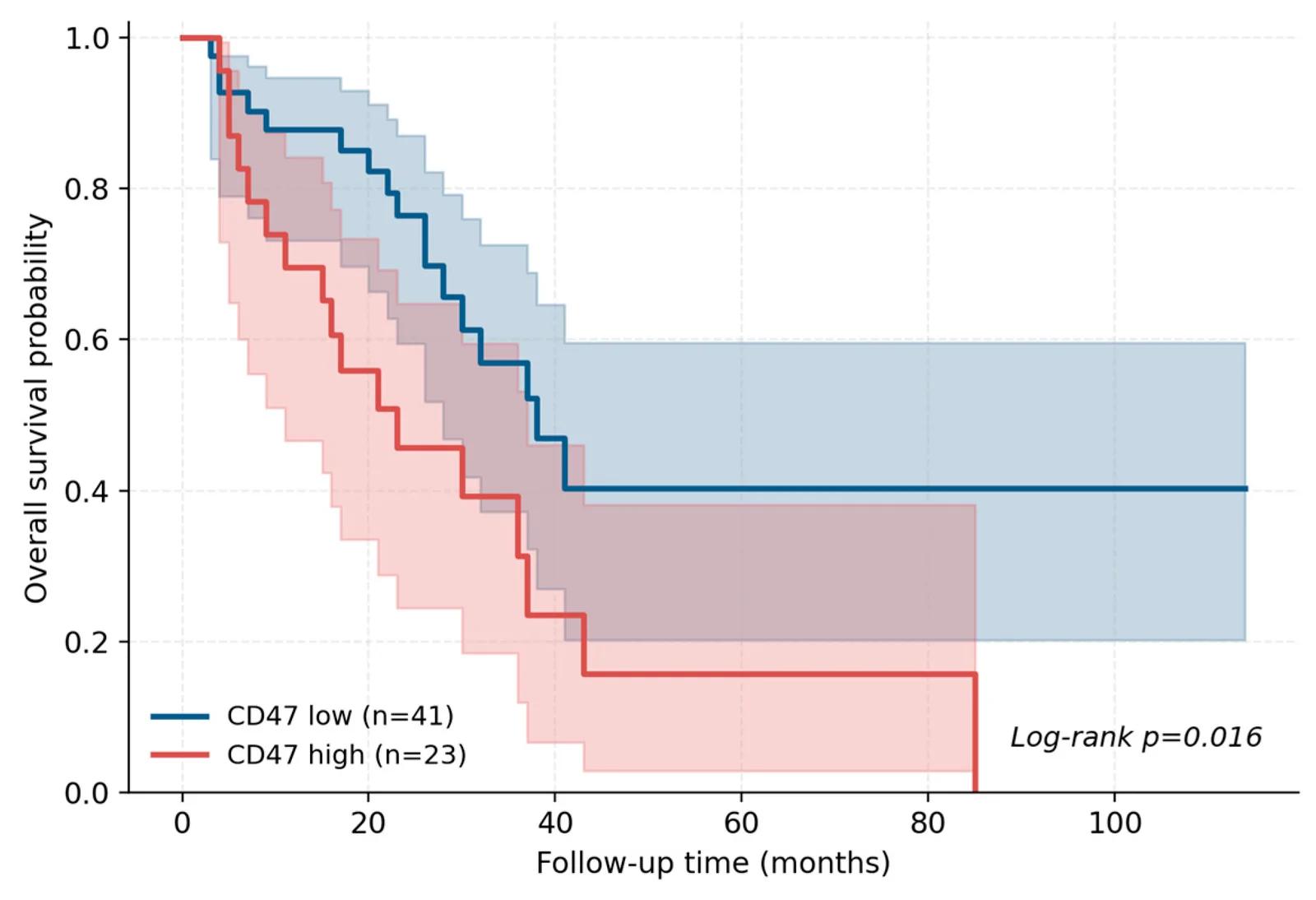
Overall survival by CD47 expression level (Kaplan–Meier curve, log-rank *p* = 0.016). Shaded areas indicate 95% confidence intervals.

**Figure 5:**
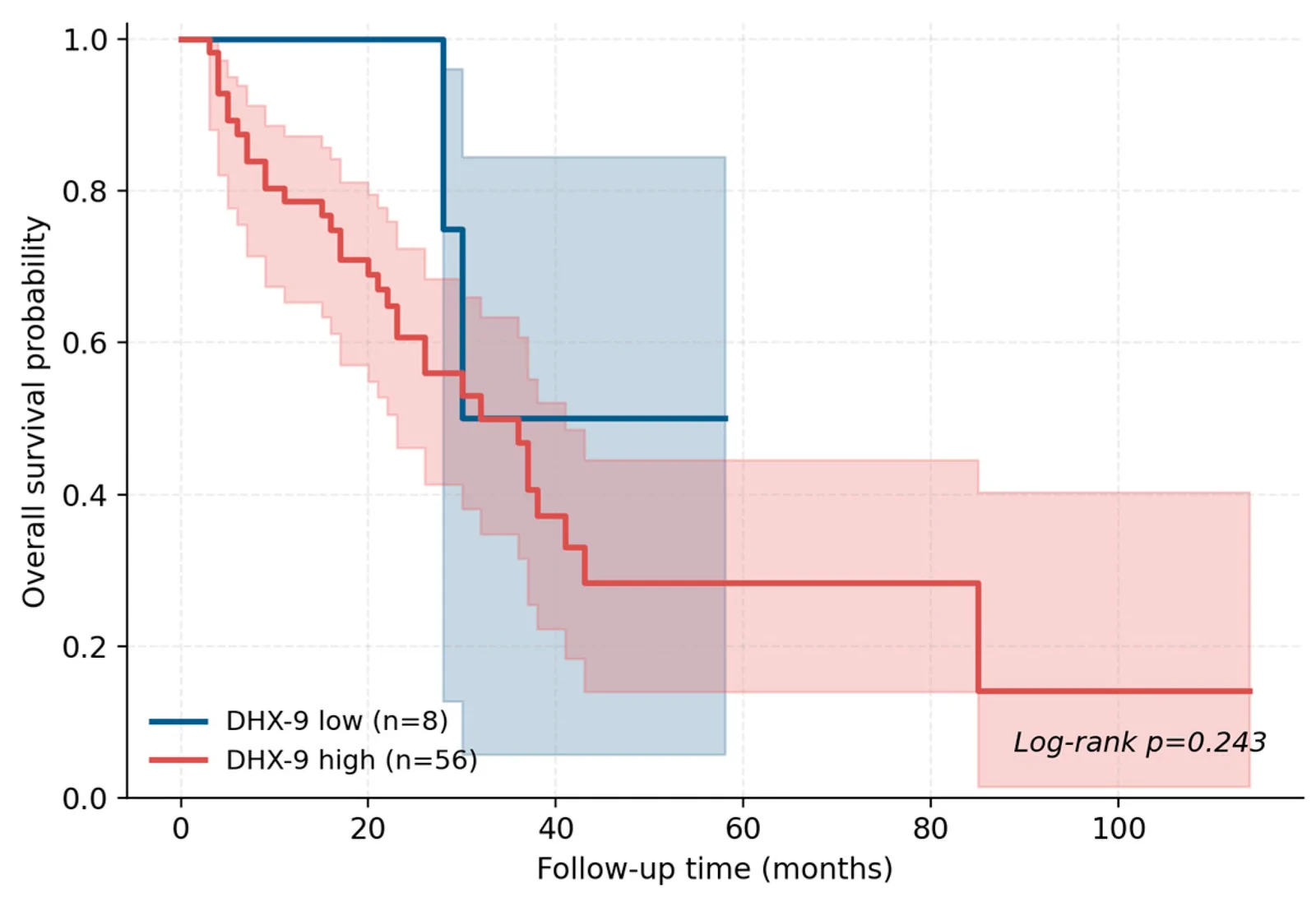
Overall survival by DHX9 expression level (Kaplan–Meier curve, log-rank *p* = 0.243). Shaded areas indicate 95% confidence intervals.

Kaplan–Meier analysis showed that high CD47 expression was associated with significantly shorter overall survival (log-rank *p* = 0.016). By contrast, overall survival did not differ significantly according to DHX9 expression (log-rank *p* = 0.243) (Figure 4 and Figure 5). When DHX9 and CD47 were modelled simultaneously as continuous staining percentages (*n* = 64, 34 events; adjusted for age, sex and metastatic presentation), higher CD47 staining remained associated with shorter overall survival (HR = 1.17 per 10% increment; 95% CI 1.04–1.32; *p* = 0.008), whereas DHX9 was not (HR = 1.11 per 10% increment; 95% CI 0.89–1.32; *p* = 0.33), consistent with the dichotomised analysis.

**Figure 6:**
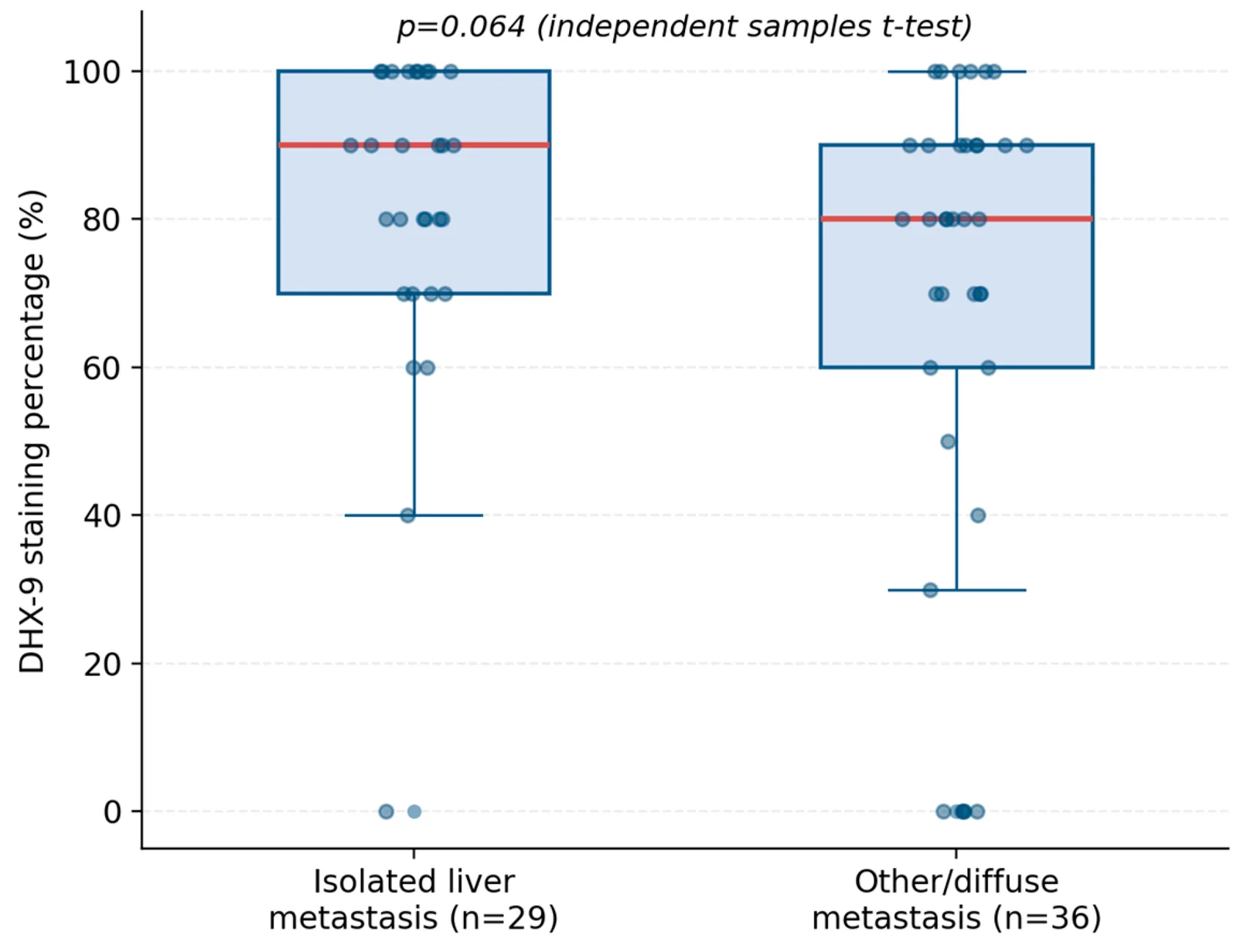
Comparison of DHX9 staining percentage between patients with and without isolated liver metastasis. Boxes indicate the interquartile range, red lines the median, and dots individual patients.

In multivariable Cox proportional hazards regression, high CD47 expression remained independently associated with shorter overall survival after adjustment for age, sex, metastatic presentation and DHX9 expression (Model 1, *n* = 64, 34 events: HR = 2.71; 95% CI 1.34–5.48; *p* = 0.006). Model 2, which additionally included RAS/RAF mutation status and HER2 positivity, was a complete-case analysis restricted to the 49 patients (23 events) with complete covariate data; the association of CD47 with overall survival remained statistically significant (HR = 2.78; 95% CI 1.02–7.59; *p* = 0.047). To distinguish the effect of additional adjustment from the effect of the reduced sample, Model 1 was re-run in this same 49-patient subset: CD47 remained independently associated with shorter overall survival (HR = 2.99; 95% CI 1.23–7.25; *p* = 0.016), suggesting that the CD47 association was not attributable to sample composition. None of the other variables, including DHX9 expression, age, sex, metastatic presentation, RAS/RAF mutation status and HER2 positivity, was independently associated with overall survival (all *p* > 0.05) (Table 4). In an exploratory subgroup analysis by metastatic site, the DHX9 staining percentage was numerically higher in patients with isolated liver metastasis than in those with other metastatic patterns, but the difference did not reach statistical significance (*p* = 0.064) (Figure 6).

## 4. Discussion

Among these 65 patients with metastatic rectal cancer, high CD47 expression was associated with shorter overall survival after adjustment. In Model 1, this association remained significant after adjustment for age, sex, metastatic presentation and DHX9 expression (HR = 2.71; 95% CI 1.34–5.48; *p* = 0.006). It also remained significant in Model 2 after further adjustment for RAS/RAF mutation status and HER2 positivity (HR = 2.78; 95% CI 1.02–7.59; *p* = 0.047). By contrast, DHX9 expression was not associated with overall survival. Expression of neither marker differed between de novo and recurrent metastatic disease or according to RAS, BRAF or HER2 status. In this exploratory analysis, CD47, but not DHX9, was associated with overall survival after adjustment for the covariates included.

CD47 has been investigated as a prognostic marker in several solid tumors. A meta-analysis of 20 studies including 4019 patients found that high CD47 expression was associated with shorter overall survival [14]. In colorectal cancer, reported positivity rates have ranged from 16% to 91%, likely reflecting methodological heterogeneity across studies [10]. High CD47 expression has been associated with advanced TNM stage, metastasis and poor differentiation [10]. In a cohort of 269 patients with advanced colorectal cancer, CD47 positivity was observed in 35% of tumors and was an independent predictor of mortality (HR = 1.75) [15]. In the same cohort, five-year survival was lower among patients with CD47-positive tumors (64.0% vs. 79.0%; *p* = 0.027) [15]. However, the independent prognostic value of CD47 has not been consistent across studies. In one cohort, high CD47 expression was associated with shorter recurrence-free survival in univariable analysis, but the association did not remain significant after adjustment [16]. Evidence specific to metastatic disease remains limited. In one report, patients with distant metastases and CD47-positive tumors had shorter overall survival than those with CD47-negative tumors (37.0 vs. 58.5 months; *p* = 0.05) [17]. The hazard ratio observed in our cohort (2.71) is consistent with these reports and with a possible prognostic role of CD47 in metastatic disease.

In colorectal cancer, DHX9 overexpression has been shown to promote tumor progression and has been associated with poor prognosis [6]. DHX9 activity has also been linked to earlier steps of disease progression, including local advancement and lymph node spread [18]. In our metastatic cohort, however, DHX9 expression was not associated with overall survival. Two factors may explain this finding. First, DHX9 was highly expressed in most tumors, leaving only eight patients in the low-expression group and thereby reducing statistical power. Second, the prognostic relevance of DHX9 may be attenuated in established metastatic disease if its biological contribution is more important during earlier stages of tumor progression. Thus, the prognostic role of DHX9 in metastatic disease remains uncertain rather than being definitively excluded.

Metastatic site is an important determinant of tumor biology and clinical outcome in colorectal cancer. The liver is the most common site of metastatic spread. Metastatic organotropism is associated with distinct molecular profiles in the primary tumor [19]. Primary tumors that metastasize to the liver differ in gene expression from those that spread to the peritoneum [19]. In our cohort, the mean DHX9 staining percentage was numerically higher in patients with isolated liver metastasis than in those with other metastatic patterns, but the difference did not reach statistical significance (*p* = 0.064). This observation should therefore be considered hypothesis-generating rather than conclusive. It raises the possibility that helicase-related pathways may be associated with hepatic tropism. However, the small subgroup size and lack of statistical significance preclude firm interpretation. CD47 expression did not vary according to metastatic site in our cohort. Larger studies are needed to determine whether either marker is associated with specific patterns of metastatic dissemination.

The prognostic significance of the timing of metastatic presentation has long been debated. Synchronous metastases are often assumed to reflect more aggressive tumor biology, although survival data remain inconsistent [20]. In advanced colorectal cancer, one study found no difference in median overall survival between synchronous and metachronous metastases despite less favorable clinicopathological features in the synchronous group [21]. A possible explanation is that prior adjuvant therapy may contribute to chemoresistance in recurrent disease, thereby offsetting the potentially more favorable biology of a metachronous presentation [21]. Consistent with these findings, overall survival did not differ between patients with de novo and recurrent metastatic disease in our cohort. DHX9 and CD47 expression categories and staining percentages were also comparable between the two groups. These findings suggest that the expression of CD47 and DHX9 does not appear to differ according to the timing of metastatic presentation.

Beyond its prognostic relevance, CD47 is also a potential therapeutic target, an important consideration in patients with metastatic disease who require systemic therapy. Antibodies targeting the CD47–SIRPα axis have been evaluated in solid tumors [9]. In colorectal cancer, the anti-CD47 antibody magrolimab was evaluated in combination with cetuximab in a phase 1b/2 trial involving 78 patients. Clinical activity was limited, with an objective response rate of 6.3% and a median overall survival of 9.5 months in KRAS wild-type disease [22]. Single-agent efficacy has not been established. Combination approaches remain under investigation [9]. Our findings suggest that CD47 may be a prognostic marker, but they do not establish its predictive value for CD47-directed therapy. Whether CD47 expression can guide patient selection for such treatment requires prospective evaluation.

## 5. Limitations

This study has several limitations. Its retrospective, single-center design and limited sample size may restrict the generalizability of the findings. The relatively short follow-up period warrants cautious interpretation of the survival estimates. Molecular data were incomplete for some patients, reducing the complete-case sample for Model 2 to 49 patients. This limitation may have reduced the precision of the effect estimates. Expression was assessed in primary tumor tissue rather than metastatic lesions, which may not capture spatial or temporal tumor heterogeneity. Only eight patients had low DHX9 expression, limiting the statistical power of analyses involving this marker. Mismatch repair or microsatellite instability status could not be evaluated because of insufficient data, and its relationship with marker expression remains to be determined. No validated cut-offs exist for DHX9 or CD47 immunohistochemistry in colorectal cancer, and our thresholds may not be generalisable to other cohorts or assays. Finally, the findings were not externally validated. The models were not adjusted for the extent of metastatic burden or for treatment variables such as metastasectomy and lines of systemic therapy; these unmeasured factors may have confounded the findings. Data on performance status and systemic treatment could not be retrieved consistently, and analysis by treatment era was not feasible; residual confounding therefore cannot be excluded. The modest number of events limited the number of covariates that could be modelled reliably, and the wider confidence interval in Model 2 reflects this reduced precision.

## 6. Conclusions

Given the retrospective, single-centre design and the limited sample size, these findings should be regarded as exploratory and hypothesis-generating rather than confirmatory. In metastatic rectal cancer, high CD47 expression was associated with shorter overall survival after adjustment for the covariates included, whereas DHX9 expression was not. The association remained significant after adjustment for the clinical and molecular variables included in the models. CD47 may have value for prognostic stratification and warrants further investigation as a potential therapeutic target. These findings require confirmation in larger prospective multicenter cohorts and validation in independent populations.

## Figures and Tables

**Table 1:** Demographic, clinical and biomarker characteristics of metastatic rectal cancer patients (*n* = 65).

Variable	Value
Age, median (range), years	63 (28–80)
Sex, *n* (%)	
Male	41 (63.1%)
Female	24 (36.9%)
BMI, mean ± SD, kg/m^2^	25.5 ± 4.3
Disease onset type, *n* (%)	
De novo metastatic	26 (40.0%)
Recurrent metastatic	39 (60.0%)
Metastasis location, *n* (%) (*n* = 65)	
Isolated liver	29 (44.6%)
Diffuse/multiple organ	22 (33.8%)
Isolated lung	7 (10.8%)
Other isolated locations	7 (10.8%)
DHX9 staining, *n* (%)	
Low	8 (12.3%)
High	57 (87.7%)
CD47 staining, *n* (%)	
Low	42 (64.6%)
High	23 (35.4%)
Median follow-up (range), months (*n* = 64)	24 (3–114)
Mortality (death), *n* (%) (*n* = 64)	34 (53.1%)
Disease progression/recurrence, *n* (%) (*n* = 65)	31 (47.7%)
BRAF mutation status known, *n*	49
KRAS mutation status known, *n*	48
NRAS mutation status known, *n*	49
HER2 status known, *n*	63

Note: The total cohort is *n* = 65. Percentages were calculated using the number of patients with available data for each variable (*n*), indicated in parentheses next to each row (e.g., the mortality rate of 53.1% was calculated over *n* = 64, excluding 1 patient with missing follow-up data).

**Table 2:** Comparison of DHX9 and CD47 expression and overall survival between de novo and recurrent metastatic disease groups.

Variable	De Novo (*n* = 26)	Recurrent (*n* = 39)	*p*
DHX9 high, *n* (%)	23 (88.5%)	34 (87.2%)	1.000
CD47 high, *n* (%)	9 (34.6%)	14 (35.9%)	1.000
DHX9 staining %, mean ± SD	76.5 ± 28.1	72.8 ± 29.4	0.610
CD47 staining %, mean ± SD	24.4 ± 30.7	24.1 ± 30.1	0.970
Overall survival (log-rank) *	*n* = 25	*n* = 39	0.681

* For the overall survival comparison, 1 de novo patient had missing follow-up data and was therefore excluded; the de novo group was analyzed as *n* = 25 in this analysis (see Figure 3 and its legend). In the expression comparisons (rows above), this patient was included, giving *n* = 26.

**Table 3:** Association between RAS/RAF/HER2 mutation or expression status and DHX9 and CD47 expression.

Marker	Positive/Mutant Rate	Association with DHX9 (*p*)	Association with CD47 (*p*)
BRAF mutation	9/49 (18.4%)	0.598	0.720
KRAS mutation	20/48 (41.7%)	0.429	0.245
NRAS mutation	0/49 (0%)	—	—
HER2 positive	4/63 (6.4%)	0.982	0.467

**Table 4:** Multivariable Cox regression analysis for overall survival.

Variable	Model 1 (*n* = 64) HR (95% CI)	*p*	Model 2 (*n* = 49) HR (95% CI)	*p*
Age (per 1 year)	1.02 (0.99–1.05)	0.319	1.01 (0.97–1.05)	0.675
Sex (Male vs. Female)	0.46 (0.21–1.01)	0.053	0.70 (0.24–2.07)	0.516
Recurrent vs. de novo metastatic	0.75 (0.35–1.59)	0.451	0.80 (0.31–2.01)	0.629
DHX9 high vs. low	2.64 (0.60–11.63)	0.200	1.93 (0.41–9.16)	0.410
CD47 high vs. low	2.71 (1.34–5.48)	0.006 *	2.78 (1.02–7.59)	0.047 *
RAS/RAF mutant	—	—	1.23 (0.46–3.31)	0.682
HER2 positive	—	—	1.02 (0.29–3.62)	0.979

* Model 1: age, sex, disease onset type (de novo vs. recurrent), DHX9 and CD47 expression; *n* = 64, 34 events, EPV = 6.8. Model 2: Model 1 covariates plus RAS/RAF mutation status and HER2 positivity; complete-case analysis, *n* = 49, 23 events, EPV = 3.3. Sensitivity: Model 1 re-run in the Model 2 subset (*n* = 49, 23 events): CD47 HR = 2.99 (95% CI 1.23–7.25), *p* = 0.016. HR: hazard ratio; CI: confidence interval; EPV: events per variable.

## Data Availability

Data supporting the findings can be obtained from the corresponding author upon reasonable request; they are not publicly accessible owing to privacy and ethical constraints.
